# Chapter 1: Biomedical Knowledge Integration

**DOI:** 10.1371/journal.pcbi.1002826

**Published:** 2012-12-27

**Authors:** Philip R. O. Payne

**Affiliations:** The Ohio State University, Department of Biomedical Informatics, Columbus, Ohio, United States of America; Whitehead Institute, United States of America; University of Maryland, Baltimore County, United States of America

## Abstract

The modern biomedical research and healthcare delivery domains have seen an unparalleled increase in the rate of innovation and novel technologies over the past several decades. Catalyzed by paradigm-shifting public and private programs focusing upon the formation and delivery of genomic and personalized medicine, the need for high-throughput and integrative approaches to the collection, management, and analysis of heterogeneous data sets has become imperative. This need is particularly pressing in the translational bioinformatics domain, where many fundamental research questions require the integration of large scale, multi-dimensional clinical phenotype and bio-molecular data sets. Modern biomedical informatics theory and practice has demonstrated the distinct benefits associated with the use of knowledge-based systems in such contexts. A knowledge-based system can be defined as an intelligent agent that employs a computationally tractable knowledge base or repository in order to reason upon data in a targeted domain and reproduce expert performance relative to such reasoning operations. The ultimate goal of the design and use of such agents is to increase the reproducibility, scalability, and accessibility of complex reasoning tasks. Examples of the application of knowledge-based systems in biomedicine span a broad spectrum, from the execution of clinical decision support, to epidemiologic surveillance of public data sets for the purposes of detecting emerging infectious diseases, to the discovery of novel hypotheses in large-scale research data sets. In this chapter, we will review the basic theoretical frameworks that define core knowledge types and reasoning operations with particular emphasis on the applicability of such conceptual models within the biomedical domain, and then go on to introduce a number of prototypical data integration requirements and patterns relevant to the conduct of translational bioinformatics that can be addressed via the design and use of knowledge-based systems.

**What to Learn in This Chapter**

- Understand basic knowledge types and structures that can be applied to biomedical and translational science;
- Gain familiarity with the knowledge engineering cycle, tools and methods that may be used throughout that cycle, and the resulting classes of knowledge products generated via such processes;
- An understanding of the basic methods and techniques that can be used to employ knowledge products in order to integrate and reason upon heterogeneous and multi-dimensional data sets; and
- Become conversant in the open research questions/areas related to the ability to develop and apply knowledge collections in the translational bioinformatics domain.

This article is part of the “Translational Bioinformatics” collection for *PLOS Computational Biology*.

## 1. Introduction

The modern biomedical research domain has experienced a fundamental shift towards integrative and translational methodologies and frameworks over the past several years. A common thread throughout the translational sciences are needs related to the collection, management, integration, analysis and dissemination of large-scale, heterogeneous biomedical data sets. However, well-established and broadly adopted theoretical and practical frameworks intended to address such needs are still largely developmental [1]–[3]. Instead, the development and execution of multi-disciplinary, translational science programs is significantly limited by the propagation of “silos” of both data and knowledge, and a paucity of reproducible and rigorously validated methods that may be used to support the satisfaction of motivating and integrative translational bioinformatics use cases, such as those focusing on the identification of expression motifs spanning bio-molecules and clinical phenotypes.

In order to provide sufficient context and scope to our ensuing discussion, we will define translational science and research per the conventions provided by the National Institutes of Health (NIH) as follows:

*“* ***Translational research*** *includes two areas of translation. One is the process of applying discoveries generated during research in the laboratory, and in preclinical studies, to the development of trials and studies in humans. The second area of translation concerns research aimed at enhancing the adoption of best practices in the community. Cost-effectiveness of prevention and treatment strategies is also an important part of translational science.”* [4]

Several recent publications have defined a ***translational research cycle***, which involves the translational of knowledge and evidence from “the bench” (e.g., laboratory-based discoveries) to “the bedside” (e.g., clinical or public health interventions informed by basic science and clinical research), and reciprocally from “the bedside” back to “the bench” (e.g., basic science studies informed by observations from the point-of-care) [5]. Within this translational cycle, Sung and colleagues [5] have defined two critical blockages that exist between basic science discovery and the design of prospective clinical studies, and subsequently between the knowledge generated during clinical studies and the provision of such evidence-based care in the clinical or public health settings. These are known as the T1 and T2 blocks, respectively. Much of the work conducted under the auspices of the NIH Roadmap initiative and more recently as part of the Clinical and Translational Science Award (CTSA) program is specifically focused on identifying approaches or policies that can mitigate these T1 and T2 blockages, and thus increase the speed and efficiency by which new biomedical knowledge can be realized in terms of improved health and patient outcomes.

The positive outcomes afforded by the close coupling of biomedical informatics with the translational sciences have been described frequently in the published literature [3], [5]–[7]. Broadly, the critical areas to be addressed by such informatics approaches relative to translational research activities and programs can be classified as belonging to one or more of the following categories:

***The management of multi-dimensional and heterogeneous data sets:*** The modern healthcare and life sciences ecosystem is becoming increasingly data centric as a result of the adoption and availability of high-throughput data sources, such as electronic health records (EHRs), research data management systems (e.g., CTMS, LIMS, Electronic Data Capture tools), and a wide variety of bio-molecular scale instrumentation platforms. As a result of this evolution, the size and complexity of data sets that must be managed and analyzed are growing at an extremely rapid rate [1], [2], [6], [8], [9]. At the same time, the data management practices currently used in most research settings are both labor intensive and rely upon technologies that have not be designed to handle such multi-dimensional data [9]–[11]. As a result, there are significant demands from the translational science community for the creation and delivery of information management platforms capable of adapting to and supporting heterogeneous workflows and data sources [2], [3], [12], [13]. This need is particularly important when such research endeavors focus on the identification of linkages between bio-molecular and phenotypic data in order to inform novel systems-level approaches to understanding disease states. Relative to the specific topic area of knowledge representation and utilization in the translational sciences, the ability to address the preceding requirements is largely predicated on the ability to ensure that semantics of such data are well understood [10], [14], [15]. This is a scenario often referred to as semantic interoperability, and requires the use of informatics-based approaches to map among various data representations, as well as the application of such mappings to support integrative data integration and analysis operations [10], [15].

***The application of knowledge-based systems and intelligent agents to enable high-throughput hypothesis generation and testing:*** Modern approaches to hypothesis discovery and testing primarily are based on the intuition of the individual investigator or his/her team to identify a question that is of interest relative to their specific scientific aims, and then carry out hypothesis testing operations to validate or refine that question relative to a targeted data set [6], [16]. This approach is feasible when working with data sets comprised of hundreds of variables, but does not scale to projects involving data sets with magnitudes on the order of thousands or even millions of variables [10], [14]. An emerging and increasingly viable solution to this challenge is the use of domain knowledge to generate hypotheses relative to the content of such data sets. This type of domain knowledge can be derived from many different sources, such as public databases, terminologies, ontologies, and published literature [14]. It is important to note, however, that methods and technologies that can allow researchers to access and extract domain knowledge from such sources, and apply resulting knowledge extracts to generate and test hypotheses are largely developmental at the current time [10], [14].

***The facilitation of data-analytic pipelines in in-silico research programs:*** The ability to execute *in-silico* research programs, wherein hypotheses are designed, tested, and validated in existing data sets using computational methods, is highly reliant on the use of data-analytic “pipelining” tools. Such pipelines are ideally able to support data extraction, integration, and analysis workflows spanning multiple sources, while capturing intermediate data analysis steps and products, and generating actionable output types [17], [18]. Such pipelines provide a number of benefits, including: 1) they support the design and execution of data analysis plans that would not be tractable or feasible using manual methods; and 2) they provide for the capture meta-data describing the steps and intermediate products generated during such data analyses. In the case of the latter benefit, the ability to capture systematic meta-data is critical to ensuring that such *in-silico* research paradigms generate reproducible and high quality results [17], [18]. There are a number of promising technology platforms capable of supporting such data-analytic “pipelining”, such as the caGrid middleware [18]. It is of note, however, that widespread use of such pipeline tools is not robust, largely due to barriers to adoption related to data ownership/security and socio-technical factors [13], [19].

***The dissemination of data, information, and knowledge generated during the course of translational science research programs:*** It is widely held that the time period required to translate a basic science discovery into clinical research, and ultimately evidence-based practice or public health intervention can exceed 15 years [2], [5], [7], [20]. A number of studies have identified the lack of effective tools for supporting the exchange of data, information, and knowledge between the basic sciences, clinical research, clinical practice, and public health practice as one of the major contributors to effective and timely translation of novel biological discoveries into health benefits [2]. A number of informatics-based approaches have been developed to overcome such translational impediments, such as web-based collaboration platforms, knowledge representation and delivery standards, public data registries and repositories [3], [7], [9], [21]. Unfortunately, the systematic and regular use of such tools and methods is generally very poor in the translational sciences, again as was the prior case, due to a combination of governance and socio-technical barriers.

At a high level, all of the aforementioned challenges and opportunities correspond to an overarching set of problem statements, as follows:

- Translational bioinformatics is defined by the presence of complex, heterogeneous, multi-dimensional data sets;
- The scope of available biomedical knowledge collections that may be applied to assist in the integration and analysis of such data is growing at a rapid pace;
- The ability to apply such knowledge collections to translational bioinformatics analyses requires an understanding of the sources of such knowledge, and methods of applying them to reasoning applications; and
- The application of knowledge collections to support integrative analyses in the translational science domain introduces multiple areas of complexity that must be understood in order to enable the optimal selection and use of such resources and methods, as well as the interpretation of results generated via such applications.

## 2. Key Definitions

**Figure 1:**
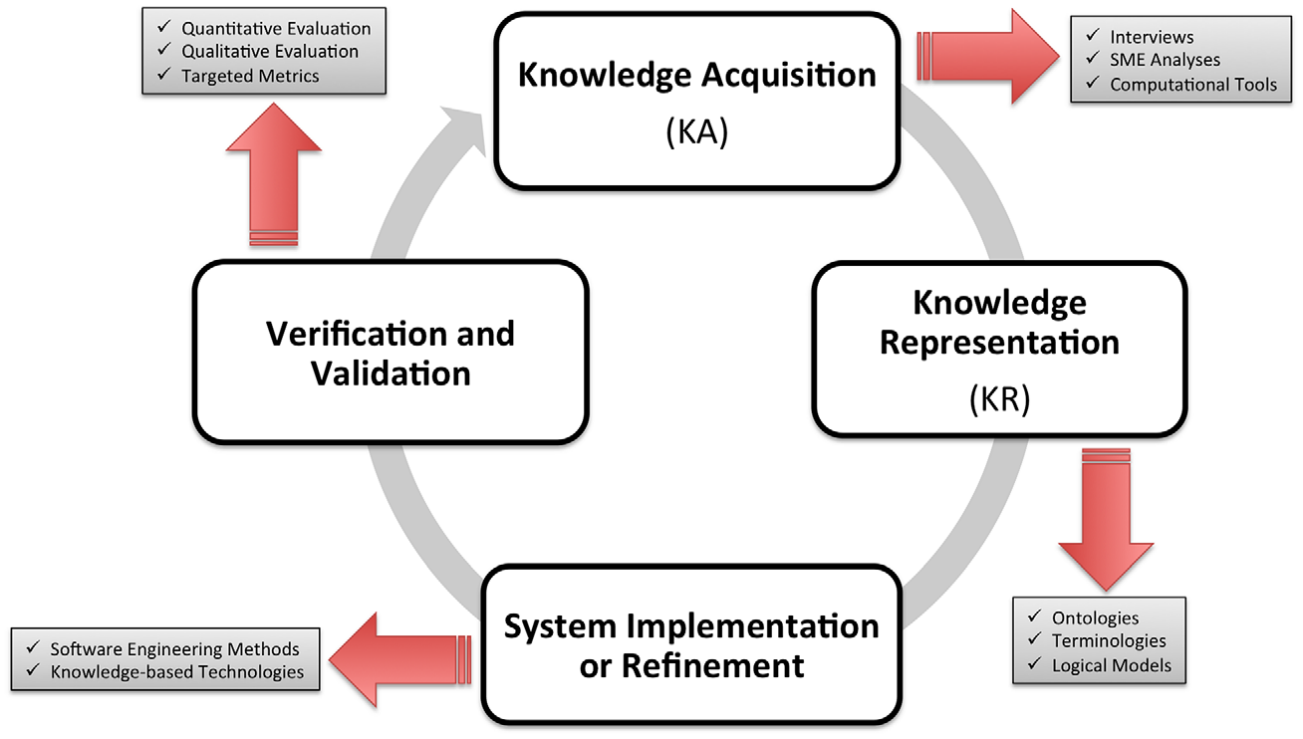
Key components of the KE process.

In the remainder of this chapter, we will introduce a set of definitions, frameworks, and methods that serve to support the foundational knowledge integration requirements incumbent to the efficient and effective conduct of translational studies. In order to provide a common understanding of key terms and concepts that will be used in the ensuing discussion, we will define here a number of those entities, using the broad context of Knowledge Engineering (KE) as a basis for such assertions. The KE process (Figure 1) incorporates multiple steps:

1. Acquisition of knowledge (KA)
2. Representation of that knowledge (KR) in a computable form
3. Implementation or refinement of knowledge-based agents or applications using the knowledge collection generated in the preceding stages
4. Verification and validation of the output of those knowledge-based agents or applications against one or more reference standards.

In the context of the final phase of the KE cycle, comparative reference standards can include expert performance measures, requirements acquired before designing the knowledge-based system, or requirements that were realized upon implementation of the knowledge-based system. In this regard, verification is the process of ensuring that the knowledge-based system meets the initial requirements of the potential end-user community. In comparison, validation is the process of ensuring that the knowledge-based system meets the realized requirements of the end-user community once a knowledge-based system has been implemented [22]. Furthermore, within the overall KE process, KA can be defined as the sub-process involving the extraction of knowledge from existent sources (e.g., experts, literature, databases, etc.) for the purpose of representing that knowledge in a computable format [23]–[28].

The KE process is intended to target three potential types of knowledge, as defined below:

- **Conceptual knowledge** is defined in the education literature as a combination of atomic units of information *and* the meaningful relationships between those units. The education literature also describes two other types of knowledge, labeled as procedural and strategic;
- **Procedural knowledge** is a process-oriented understanding of a given problem domain [29]–[32];
- **Strategic knowledge** is knowledge that is used to operationalize conceptual knowledge into procedural knowledge [31].

The preceding definitions are derived from empirical research on learning and problem-solving in complex scientific and quantitative domains such as mathematics and engineering [30], [32]. The cognitive science literature provides a similar differentiation of knowledge types, making the distinction between procedural and declarative knowledge. Declarative knowledge is synonymous with conceptual knowledge as defined above [33].

Conceptual knowledge collections are perhaps the most commonly used knowledge types in biomedicine. Such knowledge and its representation span a spectrum that includes ontologies, controlled terminologies, semantic networks and database schemas. A reoccurring focus throughout discussions of conceptual knowledge collections in the biomedical informatics domain is the process of representing conceptual knowledge in a computable form. In contrast, the process of eliciting knowledge has received less attention and reports on rigorous and reproducible methods that may be used in this area are rare. It is also important to note that in the biomedical informatics domain conceptual knowledge collections rarely exist in isolation. Instead, they usually occur within structures that contain multiple types of knowledge. For example, a knowledge-base used in a modern clinical decision support system might include: (1) a knowledge collection containing potential findings, diagnoses, and the relationships between them (*conceptual knowledge*), (2) a knowledge collection containing guidelines or algorithms used to logically traverse the previous knowledge structure (*procedural knowledge*), and (3) a knowledge structure containing application logic used to apply or operationalize the preceding knowledge collections (*strategic knowledge*). Only when these three types of knowledge are combined, it is possible to realize a functional decision support system [34].

## 3. Underlying Theoretical Frameworks

The theories that support the ability to acquire, represent, and verify or validate conceptual knowledge come from multiple domains. In the following sub-section, several of those domains will be discussed, including:

- Computational science
- Psychology and cognitive science
- Semiotics
- Linguistics

### 3.1 Computational Foundations of Knowledge Engineering

A critical theory that supports the ability to acquire and represent knowledge in a computable format is the physical symbol hypothesis. First proposed by Newell and Simon in 1981 [35], and expanded upon by Compton and Jansen in 1989 [24], the physical symbol hypothesis postulates that knowledge consists of both symbols of reality, and relationships between those symbols. The hypothesis further argues that intelligence is defined by the ability to appropriately and logically manipulate both symbols and relationships. A critical component of this the theory is the definition of what constitutes a “physical symbol system”, which Newell and Simon describe as:

*“…a set of entities, called symbols, which are physical patterns that can occur as components of another type of entity called an expression (or symbol structure). Thus, a symbol structure is composed of a number of instances (or tokens) of symbols related in some physical way (such as one token being next to another). At any instant of time the system will contain a collection of these symbol structures.”* [36]

This preceding definition is very similar to that of conceptual knowledge introduced earlier in this chapter, which leads to the observation that the computational representation of conceptual knowledge collections should be well supported by computational theory. However, as described earlier, there is not a large body of research on reproducible methods for eliciting such symbol systems. Consequently, the elicitation of the symbols and relationships that constitute a “physical symbol system”, or conceptual knowledge collection, remains a significant challenge. This challenge, in turn, is an impediment to the widespread use of conceptual knowledge-based systems.

### 3.2 Psychological and Cognitive Basis for Knowledge Engineering

At the core of the currently accepted psychological basis for KE is expertise transfer, which is the theory that humans transfer their expertise to computational systems so that those systems are able to replicate expert human performance.

One theory that helps explain the process of expertise transfer is Kelly's Personal Construct Theory (PCT). This theory defines humans as “anticipatory systems”, where individuals create templates, or constructs that allow them to recognize situations or patterns in the “information world” surrounding them. These templates are then used to anticipate the outcome of a potential action given knowledge of similar previous experiences [37]. Kelly views all people as “personal scientists” who make sense of the world around them through the use of a hypothetico-deductive reasoning system.

It has been argued within the KE literature that the constructs used by experts can be used as the basis for designing or populating conceptual knowledge collections [26]. The details of PCT help to explain how experts create and use such constructs. Specifically, Kelly's fundamental postulate is that *“a person's processes are psychologically channelized by the way in which he anticipated events.”* This is complemented by the theory's first corollary, which is summarized by his statement that:

*“Man looks at his world through transparent templates which he creates and then attempts to fit over the realities of which the world is composed… Constructs are used for predictions of things to come… The construct is a basis for making a distinction… not a class of objects, or an abstraction of a class, but a dichotomous reference axis.”*

Building upon these basic concepts, Kelly goes on to state in his Dichotomy Corollary that *“a person's construction system is composed of a finite number of dichotomous constructs.”* Finally, the parallel nature of personal constructs and conceptual knowledge is illustrated in Kelly's Organization Corollary, which states, *“each person characteristically evolves, for his convenience of anticipating events, a construction system embracing ordinal relationships between constructs”* [26], [37].

Thus, in an effort to bring together these core pieces of PCT, it can be argued that personal constructs are essentially templates applied to the creation of knowledge classification schemas used in reasoning. If such constructs are elicited from experts, atomic units of information can be defined, and the Organization Corollary can be applied to generate networks of ordinal relationships between those units. Collectively, these arguments serve to satisfy and reinforce the earlier definition of conceptual knowledge, and provide insight into the expert knowledge structures that can be targeted when eliciting conceptual knowledge.

There are also a number of cognitive science theories that have been applied to inform KE methods. Though usually very similar to the preceding psychological theories, cognitive science theories specifically describe KE within a broader context where humans are anticipatory systems who engage in frequent transfers of expertise. The cognitive science literature identifies expertise transfer pathways as an existent medium for the elicitation of knowledge from domain experts. This conceptual model of expertise transfer is often illustrated using the Hawkins model for expert-client knowledge transfer [38].

It is also important to note that at a high level, cognitive science theories focus upon the differentiation among knowledge types. As described earlier, cognitive scientists make a primary differentiation between procedural knowledge and declarative knowledge [31]. While cognitive science theory does not necessarily link declarative and procedural knowledge, an implicit relationship is provided by defining procedural knowledge as consisting of three orders, or levels. For each level, the complexity of declarative knowledge involved in problem solving increases commensurately with the complexity of procedural knowledge being used [28], [31], [39].

A key difference between the theories provided by the cognitive science and psychology domains is that the cognitive science literature emphasizes the importance of placing KA studies within appropriate context in order to account for the distributed nature of human cognition [25], [40]–[46]. In contrast, the psychology literature is less concerned with placing KE studies in context.

### 3.3 Semiotic Basis for Knowledge Engineering

**Figure 2:**
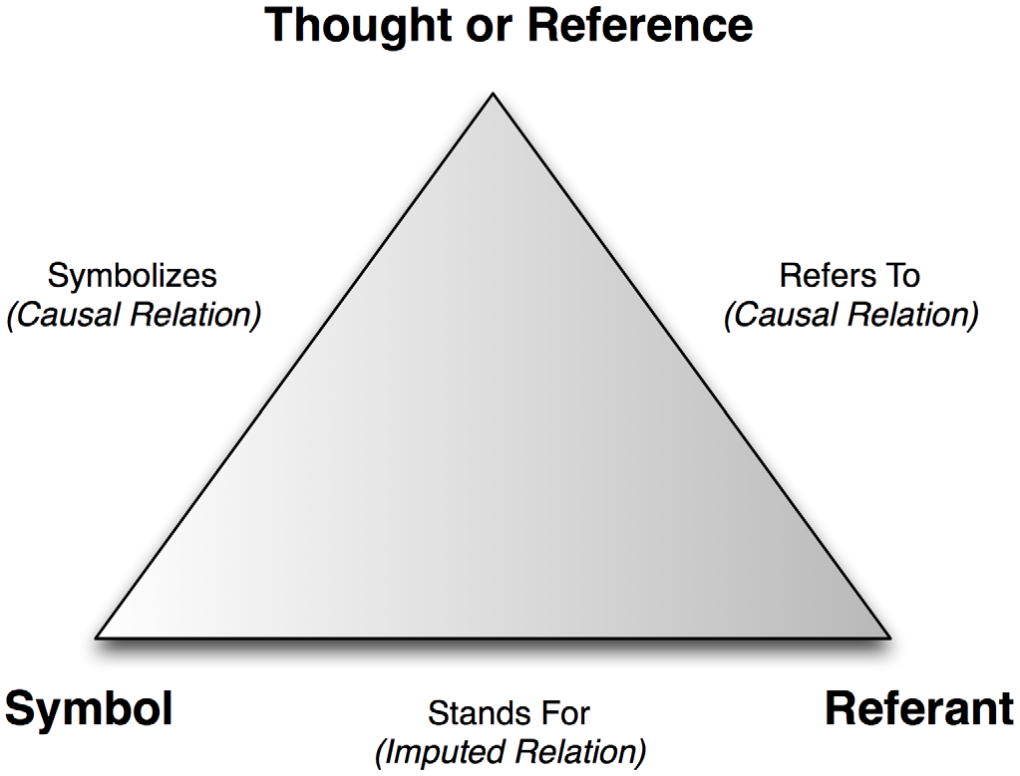
Ogden-Richards semiotic triad, illustrating the relationships between the three major semiotic-derived types of “meaning”.

Though more frequently associated with the domains of computer science, psychology and cognitive science, there are a few instances where semiotic theory has been cited as a theoretical basis for KE. Semiotics can be broadly defined as “*the study of signs, both individually and grouped in sign systems, and includes the study of how meaning is transmitted and understood*” [47]. As a discipline, much of its initial theoretical basis is derived from the domain of linguistics, and thus, has been traditionally focused on written language. However, the scope of contemporary semiotics literature has expanded to incorporate the analysis of meaning in visual presentation systems, knowledge representation models and multiple communication mediums. The basic premise of the semiotic theory of “meaning” is frequently presented in a schematic format using the Ogden-Richards semiotic triad, as shown in Figure 2 [48].

A core component of semiotic triad is the hypothesis that there exist three representational formats for knowledge, specifically:

- **Symbol**: representational artifact of a unit of knowledge (e.g., text or icons).
- **Referent**: actual unit of knowledge, which is largely a conceptual construct.
- **Thought or Reference**: unit of knowledge as actually understood by the individual or system utilizing or acting upon that knowledge.

In addition, three primary relationships are hypothesized to exist, linking the three preceding representational formats:

- **“Stands-for” imputed relation**: relationship between the symbolic representation of the knowledge and the actual unit of knowledge
- **“Refers-to” causal relation**: relationship between the actual unit of knowledge, and the unit of knowledge as understood by the individual or system utilizing or acting upon that knowledge
- **“Symbolizes” causal relation**: relationship between the unit of knowledge as understood by the individual or system utilizing or acting upon that knowledge, and the symbolic representation of the knowledge
- The strength of these relationships is usually evaluated using heuristic methods or criteria [48].

### 3.4 Linguistic Basis for Knowledge Engineering

The preceding theories have focused almost exclusively on knowledge that may be elicited from domain experts. In contrast, domain knowledge can also be extracted through the analysis of existing sources, such as collections of narrative text or databases. Sub-language analysis is a commonly described approach to the elicitation of conceptual knowledge from collections of text (e.g., narrative notes, published literature, etc.). The theoretical basis for sub-language analysis, known as sub-language theory was first described by Zellig Harris in his work concerning the nature of language usage within highly specialized domains [49]. A key argument of his sub-language theory is that language usage in such highly specialized domains is characterized by regular and reproducible structural features and grammars [49], [50]. At an application level, these features and grammars can be discovered through the application of manual or automated pattern recognition processes to large corpora of language for a specific domain. Once such patterns have been discovered, templates may be created that describe instances in which concepts and relationships between those concepts are defined. These templates can then be utilized to extract knowledge from sources of language, such as text [51]. The process of applying sub-language analysis to existing knowledge sources has been empirically validated in numerous areas, including the biomedical domain [50], [51]. Within the biomedical domain, sub-language analysis techniques have been extended beyond conventional textual language to also include sub-languages that consist of graphical symbols [52].

## 4. Knowledge Acquisition Tools and Methods

While a comprehensive review of tools and methods that may be used to facilitate the knowledge acquisition (KA) is beyond the scope of this chapter, in the following section, we will briefly summarize example cases of such techniques in order to provide a general overview of this important area of informatics research, development, and applications.

As was introduced in the preceding section, KA can be defined as the sub-process involving the extraction of knowledge from existent sources (e.g., experts, literature, databases, etc.) for the purpose of representing that knowledge in a computable format [23]–[28]. This definition also includes the verification or validation of knowledge-based systems that use the resultant knowledge collections [27]. Beyond this basic definition of KA and its relationships to KE, there are two critical characteristics of contemporary approaches to KA that should be noted, as follows:

- By convention within the biomedical informatics domain, KA usually refers to the process of eliciting knowledge specifically for use in “knowledge-bases” (KBs) that are integral to expert systems or intelligent agents (e.g., clinical decision support systems). However, a review of the literature concerned with KA beyond this domain shows a broad variety of application areas for KA, such as the construction of shared database models, ontologies and human-computer interaction models [23], [53]–[57].
- Verification and validation methods are often applied to knowledge-based systems only during the final stage of the KE process. However, such techniques are most effective when employed iteratively throughout the entire KE process. As such, they also become necessary components of the KA sub-process.

Given the particular emphasis of this chapter on the use of conceptual knowledge collections for the purpose of complex integrative analysis tasks, it is important to understand that the KA methods and tools available to support the generation of conceptual knowledge collections can be broadly divided into three complementary classes:

- **Knowledge unit elicitation**: techniques for the elicitation of atomic units of information or knowledge
- **Knowledge relationship elicitation**: techniques for the elicitation of relationships between atomic units of information or knowledge
- **Combined elicitation**: techniques that elicit both atomic units of information or knowledge, and the relationships that exist between them

There are a variety of commonly used methods that target one or more above these KA classes, as summarized below:

### 4.1 Informal and Structured Interviewing

Interviews conducted either individually or in groups can provide investigators with insights into the knowledge used by domain experts. Furthermore, they can be performed either informally (e.g., conversational exchange between the interviewer and subjects) or formally (e.g., structured using a pre-defined series of questions). The advantages of utilizing such interviewing techniques are that they require a minimal level of resources, can be performed in a relatively short time frame, and can yield a significant amount of qualitative knowledge. More detailed descriptions of interviewing techniques are provided in the methodological reviews provided by Boy [58], Morgan [59], and Wood [60].

### 4.2 Observational Studies

Ethnographic evaluations, or observational studies are usually conducted in context, with minimal researcher involvement in the workflow or situation under consideration. These observational methods generally focus on the evaluation of expert performance, and the implicit knowledge used by those experts. Examples of observational studies have been described in many domains, ranging from air traffic control systems to complex healthcare workflows [61], [62]. One of the primary benefits of such observational methods is that they are designed to minimize potential biases (e.g., Hawthorne effect [63]), while simultaneously allowing for the collection of information in context. Additional detail concerning specific observational and ethnographic field study methods can be found in the reviews provided by John [62] and Rahat [64].

### 4.3 Categorical Sorting

There are a number of categorical, or card sorting techniques, including Q-sorts, hierarchical sorts, all-in-one sorts and repeated single criterion sorts [65]. All of these techniques involve one or more subjects sorting of a group of artifacts (e.g., text, pictures, physical objects, etc.) according to criteria either generated by the sorter or provided by the researcher. The objective of such methods is to determine the reproducibility and stability of the groups created by the sorters. In all of these cases, sorters may also be asked to assign names to the groups they create. Categorical sorting methods are ideally suited for the discovery of relationships between atomic units of information or knowledge. In contrast, such methods are less effective for determining the atomic units of information or knowledge. However, when sorters are asked to provide names for their groups, this data may help to define domain-specific units of knowledge or information. Further details concerning the conduct and analysis of categorical sorting studies can be found in the review provided by Rugg and McGeorge [65].

### 4.4 Repertory Grid Analysis

Repertory grid analysis is a method based on the previously introduced Personal Construct Theory (PCT). Repertory grid analysis involves the construction of a non-symmetric matrix, where each row represents a construct that corresponds to a distinction of interest, and each column represents an element (e.g., unit of information or knowledge) under consideration. For each element in the grid, the expert completing the grid provides a numeric score using a prescribed scale (defined by a left and right pole) for each distinction, indicating the strength of relatedness between the given element-distinction pair. In many instances, the description of the distinction being used in each row of the matrix is stated differently in the left and right poles, providing a frame of reference for the prescribed scoring scale. Greater detail on the techniques used to conduct repertory grid studies can be found in the review provided by Gaines et al. [26].

### 4.5 Formal Concept Analysis

Formal concept analysis (FCA) has often been described for the purposes of developing and merging ontologies [66], [67]. FCA focuses on the discovery of “natural clusters” of entities and entity-attribute pairings [66], where attributes are similar to the distinctions used in repertory grids. Much like categorical sorting, FCA is almost exclusively used for eliciting the relationships between units of information or knowledge. The conduct of FCA studies involves two phases: (1) elicitation of “formal contexts” from subjects, and (2) visualization and exploration of resulting “concept lattices”. It is of interest to note that the “concept lattices” used in FCA are in many ways analogous to Sowa's Conceptual Graphs [68], which are comprised of both concepts and labeled relationships. The use of Conceptual Graphs has been described in the context of KR [68]–[70], as well as a number of biomedical KE instances [48], [71]–[73].

Recent literature has described the use of FCA in multi-dimensional “formal contexts” (i.e., instances where relational structures between conceptual entities cannot be expressed as a single many-valued “formal context”). One approach to the utilization of multi-dimensional “formal contexts” is the agreement context model proposed by Cole and Becker [67], which uses logic-based decomposition to partition and aggregate *n*-ary relations. This algorithmic approach has been implemented in a freely available application named “Tupleware” [74]. Additionally, “formal contexts” may be defined from existing data sources, such as databases. These “formal contexts” are discovered using data mining techniques that incorporate FCA algorithms, such as the open-source TOSCANA or CHIANTI tools. Such algorithmic FCA methods are representative examples of a sub-domain known as Conceptual Knowledge Discovery and Data Analysis (CKDD) [75]. Additional details concerning FCA techniques can be found in the reviews provided by Cimiano et al. [66], Hereth et al. [75], and Priss [76].

### 4.6 Protocol and Discourse Analysis

The techniques of protocol and discourse analysis are very closely related. Both techniques are concerned with the elicitation of knowledge from individuals while they are engaged in problem-solving or reasoning tasks. Such analyses may be performed to determine the unit of information or knowledge, and relationships between those units of information or knowledge, used by individuals performing tasks in the domain under study. During protocol analysis studies, subjects are requested to “think out loud” (i.e., vocalize internal reasoning and thought processes) while performing a task. Their vocalizations and actions are recorded for later analysis. The recordings are then codified at varying levels of granularity to allow for thematic or statistical analysis [77], [78]. Similarly, discourse analysis is a technique by which an individual's intended meaning within a body of text or some other form of narrative discourse (e.g., transcripts of a “think out loud” protocol analysis study) is ascertained by atomizing that text or narrative into discrete units of thought. These “thought units” are then subject to analyses of both the context in which they appear, and the quantification and description of the relationships between those units [79], [80]. Specific methodological approaches to the conduct of protocol and discourse analysis studies can be found in the reviews provided by Alvarez [79] and Polson et al. [78].

### 4.7 Sub-Language Analysis

Sub-language analysis is a technique for discovering units of information or knowledge, and the relationships between them within existing knowledge sources, including published literature or corpora of narrative text. The process of sub-language analysis is based on the sub-language theory initially proposed by Zellig Harris [49]. The process by which concepts and relationships are discovered using sub-language analysis is a two-stage approach. In the first stage, large corpora of domain-specific text are analyzed either manually or using automated pattern recognition techniques, in an attempt to define a number of critical characteristics, which according to Friedman et al. [50] include:

- Semantic categorization of terms used within the sub-language
- Co-occurrence patterns or constraints, and paraphrastic patterns present within the sub-language
- Context-specific omissions of information within the sub-language
- Intermingling of sub-language and general language patterns
- Usage of terminologies and controlled vocabularies (i.e., limited, reoccurring vocabularies) within the sub-language

Once these characteristics have been defined, templates or sets of rules may be established. In the second phase, the templates or rules resulting from the prior step are applied to narrative text in order to discover units of information or knowledge, and the relationships between those units. This is usually enabled by a natural language processing engine or other similar intelligent agent [81]–[85].

### 4.8 Laddering

Laddering techniques involve the creation of tree structures that hierarchically organize domain-specific units of information or knowledge. Laddering is another example of a technique that can be used to determine both units of information or knowledge and the relationships between those units. In conventional laddering techniques, a researcher and subject collaboratively create and refine a tree structure that defines hierarchical relationships *and* units of information or knowledge [86]. Laddering has also been reported upon in the context of structuring relationships between domain-specific processes (e.g., procedural knowledge). Therefore, laddering may also be suited for discovering strategic knowledge in the form of relationships between conceptual and procedural knowledge. Additional information concerning the conduct of laddering studies can be found in the review provided by Corbdridge et al. [86].

### 4.9 Group Techniques

Several group techniques for multi-subject KA studies have been reported, including brainstorming, nominal group studies, Delphi studies, consensus decision-making and computer-aided group sessions. All of these techniques focus on the elicitation of consensus-based knowledge. It has been argued that consensus-based knowledge is superior to the knowledge elicited from a single expert [27]. However, conducting multi-subject KA studies can be difficult due to the need to recruit appropriate experts who are willing to participate, or issues with scheduling mutually agreeable times and locations for such groups to meet. Furthermore, it is possible in multi-subject KA studies for a forceful or coercive minority of experts or a single expert to exert disproportionate influence on the contents of a knowledge collection [25], [27], [59], [87]. Additional detail concerning group techniques can be found in reviews provided by Gaines [26], Liou [27], Morgan [59], Roth [88], and Wood [60].

## 5. Integrating Knowledge in the Translational Science Domain

Building upon the core concepts introduced in Section 1–4, in the remainder of this chapter we will synthesize the requirements, challenges, theories, and frameworks discussed in the preceding sections, in order to propose a set of methodological approaches to the data, information, and knowledge integration requirements incumbent to complex translational science projects. We believe that it is necessary to design and execute informatics efforts in such context in a manner that incorporates tasks and activities related to: 1) the identification of major categories of information to be collected, managed and disseminated during the course of a project; 2) the determination of the ultimate data and knowledge dissemination requirements of project-related stake-holders; and 3) the systematic modeling and semantic annotation of the data and knowledge resources that will be used to address items (1) and (2).

Based upon prior surveys of the state of biomedical informatics relative to the clinical and translational science domains [3], [89], a framework that is informative to preceding design and execution pattern can be formulated. Central to this framework are five critical information or knowledge types involved in the conduct of translational science projects, as are briefly summarized in Table 1.

**Table 1:** Overview of information and knowledge types incumbent to the translational sciences.

Information or Knowledge Type	Description	Examples Sources or Types
*Individual and/or Population Phenotype*	This information type involves data elements and metadata that describe characteristics at the individual or population levels that relate to the physiologic and behavioral manifestation of healthy and disease states.	• Demographics• Clinical exam findings• Qualitative characteristics• Laboratory testing results
*Individual and/or Population Bio-markers*	This information type involves data elements and metadata that describe characteristics at the individual or population levels that relate to the bio-molecular manifestation of healthy and disease states.	• Genomic, proteomic and metabolomic expression profiles• Novel bio-molecular assays capable of measuring bio-molecular structure and function
*Domain Knowledge*	This knowledge type is comprised of community-accepted, or otherwise verified and validated [17] sources of biomedical knowledge relevant to a domain of interest. Collectively, these types of domain knowledge may be used to support multiple operations, including: 1) hypothesis development; 2) hypothesis testing; 3) comparative analyses; or 4) augmentation of experimental data sets with statistical or semantic annotations [15], [17], [125].	• Literature databases• Public or private databases containing experimental results or reference standards• Ontologies• Terminologies
*Biological Models and Technologies*	This knowledge type typically consists of: 1) empirically validated system or sub-system level models that serve to define the mechanisms by which bio-molecular and phenotypic processes and their markers/indicators interact as a network [6], [20], [124], [126]; and 2) novel technologies that enable the analysis of integrative data sets in light of such models. By their nature these tools include algorithmic or embedded knowledge sources [124], [126].	• Algorithms• Quantitative Models• Analytical “Pipelines”• Publications
*Translational Biomedical Knowledge*	Translational biomedical knowledge represents a sub-type of general biomedical knowledge that is concerned with a systems-level synthesis (i.e., incorporate quantitative, qualitative, and semantic annotations) of pathophysiologic or biophysical processes or functions of interest (e.g., pharmacokinetics, pharmacodynamics, bionutrition, etc.), and the markers or other indicators that can be used to instrument and evaluate such models.	• Publications• Guidelines• Integrative Data Sets• Conceptual Knowledge Collections

The preceding framework of information and knowledge types ultimately informs a conceptual model for knowledge integration in the translational sciences. The role of Biomedical Informatics and KE in this framework is to address the four major information management challenges enumerated earlier relative to the ability to generate ***Translational Biomedical Knowledge***, namely: 1) the collection and management of high throughput, multi-dimensional data; 2) the generation and testing of hypotheses relative to such integrative data sets; 3) the provision data analytic “pipelines”; and 4) the dissemination of knowledge collections resulting from research activities.

### 5.1 Design Pattern for Translational Science Knowledge Integration

**Figure 3:**
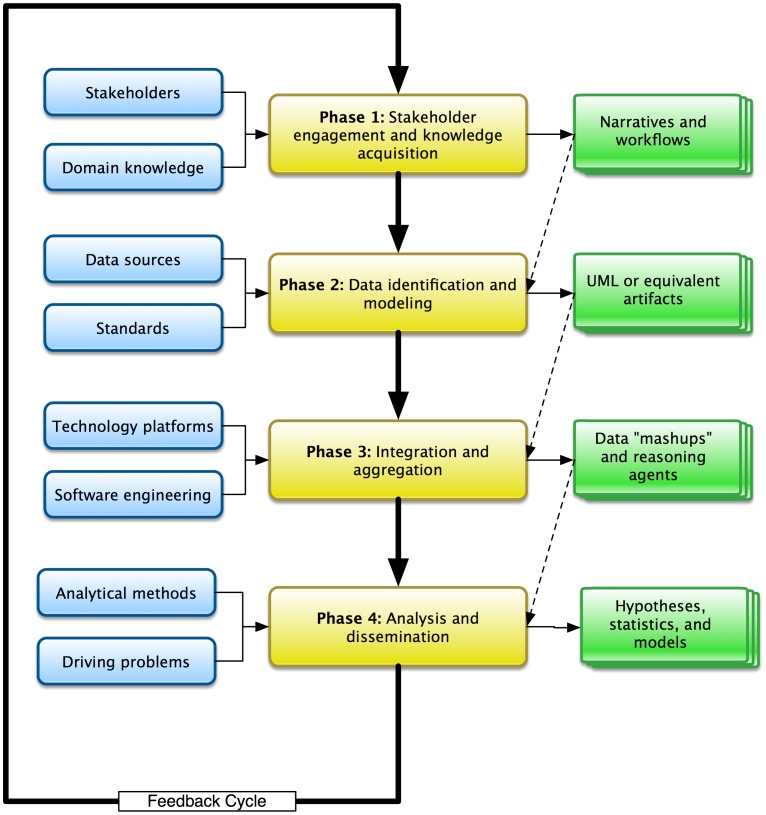
Practical model for the design and execution of translational informatics projects, illustrating major phases and exemplary input or output resources and data sets.

Informed by the conceptual framework introduced in the preceding section and illustrated in Figure 3, we will now summarize the design and execution pattern used to address such knowledge integration requirements. This design pattern can be broadly divided into four major phases that collectively define a cyclical and iterative process (which we will refer to as a ***translational research cycle***,). For each phase of the pattern, practitioners must consider both the required inputs and anticipated outputs, and their interrelationships between and across phases.

**Phase 1 - Stakeholder engagement and knowledge acquisition:** During this initial phase, key stakeholders who will be involved in the collection, management, analysis, and dissemination of project-specific data and knowledge are identified and engaged in both formal and informal knowledge acquisition, with the ultimate goal of defining the essential workflows, processes, and data sources (including their semantics). Such knowledge acquisition usually requires the use of ethnographic, cognitive science, workflow modeling, and formal knowledge acquisition techniques [14]. The results of such activities can be formalized using a thematic narratives [90]–[92] and workflow or process artifacts [92]–[94]. In some instances, it may be necessary to engage domain-specific subject matter experts (SMEs) who are not involved in a given project in order to augment available SMEs, or to validate the findings generated during such activities [14], [92].

**Phase 2 - Data identification and modeling:** Informed by the artifacts generated in Phase 1, in this phase, we focus upon the identification of specific, pertinent data sources relative to project aims, and the subsequent creation of models that encapsulate the physical and semantic representations of that data. Once pertinent data sources have been identified, we must then model their contents in an implementation-agnostic manner, an approach that is most frequently implemented using model-driven architecture techniques [95]–[99]. The results of such MDA processes are commonly recorded using the Unified Modeling Language (UML) [16], [100]–[102]. During the modeling process, it is also necessary to identify and record semantic or domain-specific annotation of targeted data structures, using locally relevant conceptual knowledge collections (such as terminologies and ontologies), in order to enable deeper, semantic reasoning concerning such data and information [16], [103], [104].

**Phase 3 - Integration and aggregation:** A common approach to the integration of heterogeneous and multi-dimensional data is the use of technology-agnostic domain or data models (per Phases 1–2), incorporating semantic annotations, in order to execute data federation operations [105] or to transform that data and load it into an integrative repository, such as a data warehouse [106]–[108]. Once the mechanisms needed to integrate such disparate data sources are implemented, it is then possible to aggregate the data for the purposes of hypothesis discovery and testing – a process that is sometimes referred to as creating a data “mashup” [109]–[115]. Data “mashups” are often created using a variety of readily available reasoners, such as those associated with the semantic web [109]–[115], which directly employ both the data models and semantic annotations created in the prior phases of the Translational Informatics Cycle, and enable a knowledge-anchored approach to such operations.

**Phase 4 - Analysis and dissemination:** In this phase of the Translational Informatics Cycle, the integrated/aggregated data and knowledge created in the preceding phases is subject to analysis. In most if not all cases, these analyses make use of domain or task specific applications and algorithms, such as those implemented in a broad number of biological data analysis packages, statistical analysis applications, and data mining tools, and intelligent agents. These types of analytical tools are used to address questions pertaining to one or more of the following four basic query or data interrogation patterns: 1) to generate hypotheses concerning relationships or patterns that serve to link variables of interest in a data set [116]; 2) to evaluate the validity hypotheses and the strength of their related data motifs, often using empirically-validated statistical tests [117], [118]; 3) to visualize complex data sets in order to facilitate human-based pattern recognition [119]–[121]; and 4) to infer and/or verify and validate quantitative models that formalize phenomena of interest identified via the preceding query patterns [122], [123].

## 6. Open Research Questions and Future Direction

**Figure 4:**
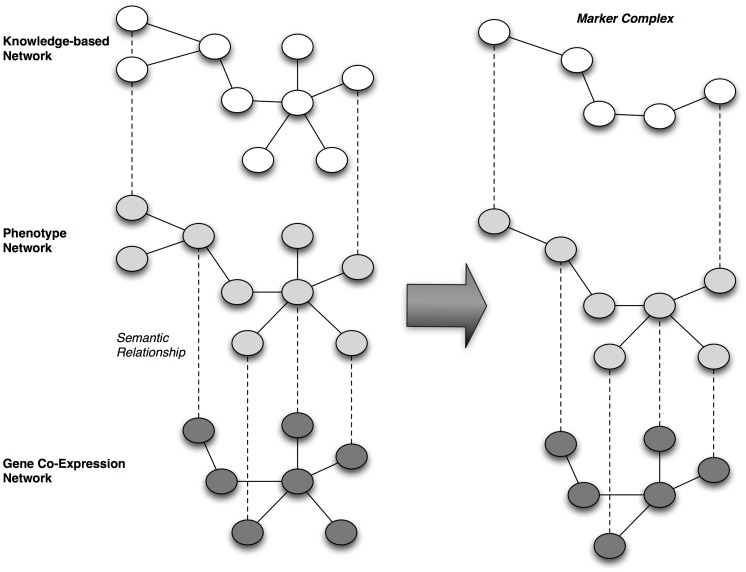
Conceptual model for the generation of multi-network complexes of markers spanning a spectrum of granularity from bio-molecules to clinical phenotypes.

As can be ascertained from the preceding review of the theoretical and practice bases for the integration of data and knowledge in the translational science domain, such techniques and frameworks have significant potential to positively impact the speed, efficacy, and impact of such research programs, and to enable novel scientific paradigms that would not otherwise be tractable. However, there are a number of open and ongoing research and development questions being addressed by the biomedical informatics community relative to such approaches that should be noted:

**Dimensionality and granularity:** the majority of knowledge integration techniques being designed, evaluated, and applied relative to the context of the translational science domain target low-order levels of dimensionality (e.g., the integration of data and knowledge corresponding to a single type, per the definitions set forth in Table 1). However, many translational science problem spaces require reasoning across knowledge-types and data granularities (e.g., multidimensional data and knowledge collections). The ability to integrate and reason upon data in a knowledge-anchored manner that addresses such multi-dimensional context remains an open area of research. Many efforts to address this gap in knowledge and practice rely upon the creation of semantically typed “vertical” linkages spanning multiple integrative knowledge networks, as is illustrated in Figure 4.

**Scalability:** Similar to the challenge of dimensionality and granularity, the issue of scalability of knowledge integration methods also remains an open area of research and development. Specifically, a large number of available knowledge integration techniques rely upon semi-automated or human-mediated methods or activities, which significantly curtail the scalability of such approaches to large-scale problems. Much of the research targeting this gap in knowledge and practice has focused on the use of artificial intelligence and semantic-reasoning technologies to enable the extraction, disambiguation, and application of conceptual knowledge collections.

**Reasoning and visualization:** Once knowledge and data have been aggregated and made available for hypothesis discovery and testing, the ability to reason upon and visualize such “mashups” is highly desirable. Current efforts to provide reusable methods of doing so, such as the tools and technologies provided by the semantic web community, as well as visualization techniques being explored by the computer science and human-computer interaction communities, hold significant promise in addressing such needs, but are still largely developmental.

**Applications of knowledge-based systems for** ***in-silico*** **science paradigms:** As has been discussed throughout this collection, a fundamental challenge in Translational Bioinformatics is the ability to both ask and answer the full spectrum of questions possible given a large-scale and multi-dimensional data set. This challenge is particularly pressing at the confluence of high-throughput bio-molecular measurement methods and the translation of the findings generated by such approaches to clinical research or practice. Broadly speaking, overcoming this challenge requires a paradigm that can be described as *in-silico* science, in which informatics methods are applied to generate and test integrative hypotheses in a high-throughput manner. Such techniques require the development and use of novel KA and KR methods and structures, as well as the design and verification/validation of knowledge-based systems targeting the aforementioned intersection point. There are several exemplary instances of investigational tools and projects targeting this space, including RiboWeb, BioCyc, and a number of initiatives concerned with the modeling and analysis of complex biological systems [6], [113], [114], [120], [124]. In addition, there are a number of large-scale conceptual knowledge collections focusing on this particular area that can be explored as part of the repositories maintained and curated by the National Center for Biomedical Ontologies (NCBO). However, broadly accepted methodological approaches and knowledge collections related to this area generally remain developmental.

## 7. Summary

As was stated at the outset of this chapter, our goals were to review the basic theoretical frameworks that define core knowledge types and reasoning operations with particular emphasis on the applicability of such conceptual models within the biomedical domain, and to introduce a number of prototypical data integration requirements and patterns relevant to the conduct of translational bioinformatics that can be addressed via the design and use of knowledge-based systems. In doing so, we have provided:

- Definitions of the **basic knowledge types and structures** that can be applied to biomedical and translational research;
- An overview of the **knowledge engineering cycle**, and the products generated during that cycles;
- Summaries of basic **methods, techniques, and design patterns that can be used to employ knowledge products** in order to integrate and reason upon heterogeneous and multi-dimensional data sets; and
- An introduction to the **open research questions and areas related to the ability to apply knowledge collections** and knowledge-anchored reasoning processes across multiple networks or knowledge collections.

Given that the translational bioinformatics is defined by the presence of complex, heterogeneous, multi-dimensional data sets, and in light of the growing volume of biomedical knowledge collections, the ability to apply such knowledge collections to biomedical data sets requires an understanding of the sources of such knowledge, and methods of applying them to reasoning applications. Ultimately, these approaches introduce both significant opportunities to advance the state of translational science, while simultaneously adding areas of complexity to the design of translational bioinformatics studies, including the methods needed to reason in an integrative manner across multiple networks or knowledge constructs. As such, these theories, methods, and frameworks offer significant benefits as well as a number of exciting and ongoing areas of biomedical informatics research and development.

## 8. Exercises

**Instructions:** Read the following motivating use case and then perform the tasks described in each question. The objective of this exercise is to demonstrate how available and open-access knowledge discovery and reasoning tools can be used to satisfy the information needs incumbent to biomedical knowledge integration needs commonly encountered in the clinical and translational research environment.

**Use Case:** *The ability to identify potentially actionable phenotype-to-biomarker relationships is of critical importance in the translational science domain. In the specific context of integrative cancer research, it is regularly the case that structural and functional relationships between genes, gene products, and clinical phenotypes are used to design and evaluate diagnostic and therapeutic approaches to targeted disease states. Large volumes of domain specific conceptual knowledge related to such hypothesis generation processes can be found in publically available literature corpora and ontologies*.

1. **Task One:** Select a specific cancer and perform a search for a collection of recent literature available with full free text via PubMed Central (the resulting corpora should contain 5 manuscripts published within the last three years, selected based upon their publication dates beginning with the most recent articles/manuscripts). Download the free text for each such article.
2. **Task Two:** For each full text article in the corpora established during Task One, semantically annotate genes, gene products, and clinical phenotype characteristics as found in the Abstract, Introduction, and Conclusion (or equivalent) sections using applicable Gene Ontology (GO) concepts, using the NCBO annotator found at: http://bioportal.bioontology.org/annotator)
3. **Task Three:** Identify the top 10 most frequently occurring Gene Ontology (GO) concepts found in your annotations, per Task Two. For each such concept, perform a search of PubMed Central for articles in which both the appropriate terms describing the cancer selected in Task One as well as these concepts co-occur. For the top 5 (most recent) articles retrieved via each search, retrieve the associate abstract for subsequent analysis
4. **Task Four:** Using the NCBO Ontology Recommender (http://bioportal.bioontology.org/recommender), analyze each of the abstracts retrieved in Task Three to determine the optimal ontology for annotating those abstracts, noting the top “recommended” ontology for each such textual resource.
5. **Task Five:** For each abstract identified in Step Three, again using the NCBO annotator (found at: http://bioportal.bioontology.org/annotator), annotate those abstracts using the recommended ontologies identified in Step Four (selecting only those ontologies that are also reflects in the NLM's UMLS). Then, for the top 2–3 phenotypic (e.g., clinically relevant) concepts identified via that annotation process, use the UMLS UTS (https://uts.nlm.nih.gov/) in order to identify potential phenotype-genotype pathways linking such phenotypic concepts and the genes or gene products identified in Task Two. Please note that performing this task will require exploring multiple relationship types reflected in the UMLS metathesaurus (documentation concerning how to do perform such a search can be found here: http://www.ncbi.nlm.nih.gov/books/NBK9684/).

Answers to the Exercises can be found in Text S1.

**Further Reading**

- Brachman RJ, McGuinness DL (1988) Knowledge representation, connectionism and conceptual retrieval. Proceedings of the 11th annual international ACM SIGIR conference on research and development in information retrieval. Grenoble, France: ACM Press.
- Campbell KE, Oliver DE, Spackman KA, Shortliffe EH (1998) Representing thoughts, words, and things in the UMLS. J Am Med Inform Assoc 5: 421–431.
- Compton P, Jansen R (1990) A philosophical basis for knowledge acquisition Knowledge Acquisition 2: 241–257.
- Gaines BR (1989) Social and cognitive processes in knowledge acquisition. Knowledge Acquisition 1: 39–58.
- Kelly GA (1955) The psychology of personal constructs. New York: Norton. 2 v. (1218).
- Liou YI (1990) Knowledge acquisition: issues, techniques, and methodology. Orlando, Florida, United States: ACM Press. pp. 212–236.
- McCormick R (1997) Conceptual and procedural knowledge. International Journal of Technology and Design Education 7: 141–159.
- Newell A, Simon HA (1981) Computer science as empirical inquiry: symbols and search. In: Haugeland J, editor. Mind design. Cambridge: MIT Press/Bradford Books. pp. 35–66.
- Patel VL, Arocha JF, Kaufman DR (2001) A primer on aspects of cognition for medical informatics. J Am Med Inform Assoc 8: 324–343.
- Preece A (2001) Evaluating verification and validation methods in knowledge engineering. Micro-Level Knowledge Management: 123–145.
- Zhang J (2002) Representations of health concepts: a cognitive perspective. J Biomed Inform 35: 17–24.

**Glossary**

- **Data:** factual information (as measurements or statistics) used as a basis for reasoning, discussion, or calculation [127]
- **Information:** knowledge obtained from investigation, study, or instruction [127]
- **Knowledge:** the circumstance or condition of apprehending truth or fact through reasoning [127]
- **Knowledge engineering:** a branch of artificial intelligence that emphasizes the development and use of expert systems [128]
- **Knowledge acquisition:** the act of acquiring knowledge
- **Knowledge representation:** the symbolic formalization of knowledge
- **Conceptual knowledge**: knowledge that consists of atomic units of information and meaningful relationships that serve to interrelate those units.
- **Strategic knowledge:** knowledge used to infer procedural knowledge from conceptual knowledge.
- **Procedural knowledge:** knowledge that is concerned with a problem-oriented understanding of how to address a given task or activity.
- **Terminology:** the technical or special terms used in a business, art, science, or special subject [128]
- **Ontology:** a rigorous and exhaustive organization of some knowledge domain that is usually hierarchical and contains all the relevant entities and their relations [128]
- **Multi-dimensional data:** data spanning multiple levels or context of granularity or scope while maintaining one or more common linkages that span such levels
- **Motif:** a reproducible pattern
- **Mashup:** a combination of multiple, heterogeneous data or knowledge sources in order to create an aggregate collection of such elements or concepts.
- **Intelligent agent:** a software agent that employs a formal knowledge-base in order to replicate expert performance relative to problem solving in a targeted domain.
- **Clinical phenotype:** the observable physical and biochemical characteristics of an individuals that serve to define clinical status (e.g., health, disease)
- **Biomarker:** a bio-molecular trait that can be measure to assess risk, diagnosis, status, or progression of a pathophysiologic or disease state.

## Supporting Information

Text S1 Answers to Exercises
(DOCX)
