# Supplementary material for: Chapter 1: Biomedical Knowledge Integration
Source: PLoS Comput Biol. 2012 Dec 27;8(12):e1002826. doi: 10.1371/journal.pcbi.1002826 (PMC3531314; doi:10.1371/journal.pcbi.1002826)
Supplement: Text S1 — Answers to Exercises (DOCX) [file pcbi.1002826.s001.docx]

## Exercises WITH Responses

### (Please note that responses are exemplary, and will vary based upon the specific concepts/variables selected by respondents when completing the tasks)

**Instructions:** Read the following motivating use case and then perform the tasks described in each question. The objective of this exercise is to demonstrate how available and open-access knowledge discovery and reasoning tools can be used to satisfy the information needs incumbent to biomedical knowledge integration needs commonly encountered in the clinical and translational research environment.

**Use Case:** *The ability to identify potentially actionable phenotype-to-biomarker relationships is of critical importance in the translational science domain. In the specific context of integrative cancer research, it is regularly the case that structural and functional relationships between genes, gene products, and clinical phenotypes are used to design and evaluate diagnostic and therapeutic approaches to targeted disease states. Large volumes of domain specific conceptual knowledge related to such hypothesis generation processes can be found in publically available literature corpora and ontologies.*

1. **Task One:** Select a specific cancer and perform a search for a collection of recent literature available with full free text via PubMed Central (the resulting corpora should contain 5 manuscripts published within the last three years, selected based upon their publication dates beginning with the most recent articles/manuscripts). Download the free text for each such article.

**Example results:**

Search (as submitted to <http://www.ncbi.nlm.nih.gov/pmc/>):

"chronic lymphocytic leukaemia"[All Fields] OR "leukemia, lymphocytic, chronic, b-cell"[MeSH Terms] OR ("leukemia"[All Fields] AND "lymphocytic"[All Fields] AND "chronic"[All Fields] AND "b-cell"[All Fields]) OR "b-cell chronic lymphocytic leukemia"[All Fields] OR ("chronic"[All Fields] AND "lymphocytic"[All Fields] AND "leukemia"[All Fields]) OR "chronic lymphocytic leukemia"[All Fields]

Results (5 most recent manuscripts, indicated by PMCIDs):

1. PMC3459001
2. PMC3462188
3. PMC3462182
4. PMC3460894
5. PMC3460853
6. **Task Two:** For each full text article in the corpora established during Task One, semantically annotate genes, gene products, and clinical phenotype characteristics as found in the Abstract, Introduction, and Conclusion (or equivalent) sections using applicable Gene Ontology (GO) concepts, using the NCBO annotator found at: <http://bioportal.bioontology.org/annotator>)

**Example results:**

Abstract from PMC3459001:

Six volunteers experienced severe inflammatory response during the Phase I clinical trial of a monoclonal antibody that was designed to stimulate a regulatory T cell response. Soon after the trial began, each volunteer experienced a “cytokine storm”, a dramatic increase in cytokine concentrations. The monoclonal antibody, TGN1412, raised serum concentrations of both pro- and anti-inflammatory cytokines το very hiγh values during the first day, while lymphocyte and monocyte concentrations plummeted. Because the subjects were healthy and had no prior indications of immune deficiency, this event provided an unusual opportunity to study the dynamic interactions of cytokines and other measured parameters. Here, the response histories of nine cytokines have been modeled by a set of linear ordinary differential equations. A general search procedure identifies parameters of the model, whose response fits the data well during the five-day measurement period. The eighteenth-order model reveals plausible cause-and-effect relationships among the cytokines, showing how each cytokine induces or inhibits other cytokines. It suggests that perturbations in IL2, IL8, and IL10 have the most significant inductive effect, while IFN-γ and IL12 have the greatest inhibiting effect on other cytokine concentrations. Although TNF-α is a major pro-inflammatory factor, IFN-γ and three other cytokines have faster initial and median response to TGN1412 infusion. Principal-component analysis of the data reveals three clusters of similar cytokine responses: [TNF-α, IL1, IL10], [IFN-γ, IL2, IL4, IL8, and IL12], and [IL6]. IL1, IL6, IL10, and TNF-α have the highest degree of variability in response to uncertain initial conditions, exogenous effects, and parameter estimates. This study illuminates details of a cytokine storm event, and it demonstrates the value of linear modeling for interpreting complex, coupled biological system dynamics from empirical data.

Annotation Results:

| **Term** | **Ontology** | **Type** | **Context** | **Matched Term** | **Matched Ontology** |
| --- | --- | --- | --- | --- | --- |
| immunoglobulin complex, circulating | Gene Ontology | Direct | cytokine concentrations. The monoclonal antibody, TGN1412, raised serum concentrations | immunoglobulin complex, circulating | Gene Ontology |
| immunoglobulin complex, circulating | Gene Ontology | Direct | trial of a monoclonal antibody that was designed to | immunoglobulin complex, circulating | Gene Ontology |
| inflammatory response | Gene Ontology | Direct | Six volunteers experienced severe inflammatory response during the Phase I | inflammatory response | Gene Ontology |
| cell | Gene Ontology | Direct | stimulate a regulatory T cell response. Soon after the | cell | Gene Ontology |

1. **Task Three:** Identify the top 10 most frequently occurring Gene Ontology (GO) concepts found in your annotations, per Task Two. For each such concept, perform a search of PubMed Central for articles in which both the appropriate terms describing the cancer selected in Task One as well as these concepts co-occur. For the top 5 (most recent) articles retrieved via each search, retrieve the associate abstract for subsequent analysis

**Example results:**

Search (as submitted to <http://www.ncbi.nlm.nih.gov/pmc/>):

(("immunoglobulins"[MeSH Terms] OR "immunoglobulins"[All Fields] OR "immunoglobulin"[All Fields]) AND complex[All Fields] AND circulating[All Fields]) AND ("chronic lymphocytic leukaemia"[All Fields] OR "leukemia, lymphocytic, chronic, b-cell"[MeSH Terms] OR ("leukemia"[All Fields] AND "lymphocytic"[All Fields] AND "chronic"[All Fields] AND "b-cell"[All Fields]) OR "b-cell chronic lymphocytic leukemia"[All Fields] OR ("chronic"[All Fields] AND "lymphocytic"[All Fields] AND "leukemia"[All Fields]) OR "chronic lymphocytic leukemia"[All Fields])

Results (5 most recent manuscripts, indicated by PMCIDs):

1. PMC3447925
2. PMC3458409
3. PMC3445594
4. PMC3447344
5. PMC3460535

Abstract from PMC3447925:

Although tumor-associated macrophages (TAMs) are involved in tumor growth and metastasis, the mechanisms controlling their pro-tumoral activities remain largely unknown. The transcription factor c-MYC has been recently shown to regulate *in vitro* human macrophage polarization and be expressed in macrophages infiltrating human tumors. In this study, we exploited the predominant expression of LysM in myeloid cells to generate *c-Myc^fl/fl^ LysM^cre/+^* mice, which lack *c-Myc* in macrophages, to investigate the role of macrophage c-MYC expression in cancer. Under steady-state conditions, immune system parameters *in c-Myc^fl/fl^ LysM^cre/+^* mice appeared normal, including the abundance of different subsets of bone marrow hematopoietic stem cells, precursors and circulating cells, macrophage density, and immune organ structure. In a model of melanoma, however, TAMs lacking *c-Myc* displayed a delay in maturation and showed an attenuation of pro-tumoral functions (e.g., reduced expression of VEGF, MMP9, and HIF1α) that was associated with impaired tissue remodeling and angiogenesis and limited tumor growth in *c-Myc^fl/fl^ LysM^cre/+^* mice. Macrophage *c-Myc* deletion also diminished fibrosarcoma growth. These data identify *c-Myc* as a positive regulator of the pro-tumoral program of TAMs and suggest *c-Myc* inactivation as an attractive target for anti-cancer therapy.

1. **Task Four:** Using the NCBO Ontology Recommender (<http://bioportal.bioontology.org/recommender>), analyze each of the abstracts retrieved in Task Three to determine the optimal ontology for annotating those abstracts, noting the top “recommended” ontology for each such textual resource.

Results for Abstract from PMC3447925:

| **Rank** | **Ontology** | **Terms Matched** |
| --- | --- | --- |
| 1 | NCI Thesaurus | 285 of 89129 |
| 2 | Zebrafish anatomy and development | 47 of 2779 |
| 3 | Bone Dysplasia Ontology | 48 of 13918 |
| 4 | Teleost Anatomy Ontology | 45 of 3039 |
| 5 | SNOMED Clinical Terms | 157 of 395036 |

… (Results truncated)

1. **Task Five:** For each abstract identified in Step Three, again using the NCBO annotator (found at: <http://bioportal.bioontology.org/annotator>), annotate those abstracts using the recommended ontologies identified in Step Four (selecting only those ontologies that are also reflects in the NLM’s UMLS). Then, for the top 2-3 phenotypic (e.g., clinically relevant) concepts identified via that annotation process, use the UMLS UTS (<https://uts.nlm.nih.gov/>) in order to identify potential phenotype-genotype pathways linking such phenotypic concepts and the genes or gene products identified in Task Two. Please note that performing this task will require exploring multiple relationship types reflected in the UMLS metathesaurus (documentation concerning how to do perform such a search can be found here: <http://www.ncbi.nlm.nih.gov/books/NBK9684/>).

Results will vary based upon respondent search criteria.
